# Association Between Body Mass Index and Permanent Pacemaker Implantation After Transcatheter Aortic Valve Replacement (TAVR) with Edwards SAPIEN™ 3 TAVR Valves: A Single-Center Experience

**DOI:** 10.7759/cureus.5142

**Published:** 2019-07-15

**Authors:** Mansoor Ahmad, Jay N Patel, Brian L Loc, Sharath C Vipparthy, Chirag Divecha, Pablo X Barzallo, Minchul Kim, Timir Baman, Marco Barzallo, Sudhir Mungee

**Affiliations:** 1 Internal Medicine, University of Illinois College of Medicine at Peoria, Peoria, USA; 2 Cardiology, University of Illinois College of Medicine at Peoria, Peoria, USA

**Keywords:** bmi, permanent pacemaker, transcatheter aortic valve replacement (tavr)

## Abstract

Background: Transcatheter aortic valve replacement (TAVR) can be complicated with a high-degree atrioventricular block requiring a permanent pacemaker (PPM) in 5% - 25% of patients. Association between body mass index (BMI) and pacemaker implantation has not been extensively studied. We compared standard BMI classes with the odds of requiring a PPM implantation in patients undergoing TAVR with Edwards SAPIEN™ 3 valves (ESV3) (Edwards Lifesciences, Irvine, CA, USA).

Methods: Our study involved a single-center retrospective cohort analysis of 449 patients undergoing TAVR from December 2012 to April 2018. First, we excluded patients with a TAVR procedure done with valves other than the ESV3 (127 patients). Second, patients with a prior PPM or an implantable cardioverter-defibrillator (37 patients) were excluded. Finally, patients with an aborted procedure and surgical conversion were excluded (16 patients). The final sample size was 269. The primary outcome was pacemaker implantation. Statistical analysis was done using the Chi-square test, T-test, and adjusted logistic regression.

Results: Of the 269 patients (50.6% males; mean age of 79.5 ± 8.7 years; mean Society of Thoracic Surgeons (STS) score: 6.2), pacemaker implantation was performed in 17 patients (6.3%). Time to pacemaker implantation was 1.3 days. Patients were divided into four categories based on their BMI: as underweight (BMI < 25; 67 patients), normal (BMI: 25 to ≤ 30; 87 patients), overweight (BMI: 30 to ≤ 35; 60 patients), and obese (BMI ≥ 35; 55 patients). Pacemaker implantation was significantly higher in patients with a BMI of > 30 (13 vs. 4, p = 0.037). After logistic linear regression, the odds of getting a PPM after TAVR were significantly higher in patients who were overweight (odds ratio (OR): 12.77, p = 0.024; confidence interval (CI): 1.39 - 17.25) and obese (OR: 15.02, p = 0.036, CI: 1.19 - 19.92).

Conclusions: Our study demonstrates that increased BMI is a possible risk factor for a high-degree atrioventricular block in patients receiving ESV3.

## Introduction

Transcatheter aortic valve replacement (TAVR) is rapidly becoming the standard of care for the treatment of severe aortic stenosis. Several studies have focused on determining factors associated with TAVR complications. One notable complication is a complete heart block and the requirement for a permanent pacemaker (PPM). The post-TAVR need for PPM placement has a significant impact on overall cost, morbidity, and mortality [1].

Atrioventricular conduction block in TAVR is likely caused by a direct injury of the His bundle, given its close relation to the membranous septum and native aortic valve [2-3]. A preexisting right bundle branch block (RBBB), depth of implantation, use of a self-expanding valve (SEV), prosthesis to the left ventricular outflow tract (LVOT) diameter ratio, male gender, prolonged partial response (PR) interval, and a left anterior hemiblock have been identified as some predictors for PPM implantation in multiple studies [4-8].

Body mass index (BMI) as a predictor of PPM insertion post-TAVR has not been extensively studied. Registry data and observational studies fail to demonstrate significant BMI differences in PPM and no PPM cohorts post-TAVR [1, 9-10]. One such National (Nationwide) Inpatient Sample (NIS) database study of 6,778 obese TAVR patients had a 13% post-procedure requirement of PPM [10], which is similar to the overall incidence of PPM post-TAVR in 2015 (12%) as reported in the 2016 Annual Report of the Transcatheter Valve Therapy Registry [11].

Our goal was to study the impact of BMI on PPM implantation among our patients who underwent TAVR with the Edwards SAPIEN™ 3 valves (ESV3) over a five-year study period.

The abstract of this study was published as a poster in 2019 annual conference by Society for Cardiovascular Angiography and Interventions [12].

## Materials and methods

Patient population and study design

We utilized a retrospective chart review of 449 patients who received TAVR at OSF Saint Francis Medical Center between December 2012 and April 2018. First, we excluded patients with TAVR procedure done with valves other than ESV3 (127 patients). Second, patients with prior PPM or implantable cardioverter-defibrillator (37 patients) were excluded. Finally, patients with an aborted procedure and surgical conversion were excluded (16). The final sample size was 269.

Institutional Review Board approval was obtained from the office of Human Research at the University of Illinois Chicago, at Peoria, IL. Considering the retrospective nature of this study, a consent waiver was approved. All patients undergoing TAVR were deemed as intermediate or high-risk for SAVR by the local cardiothoracic surgery team based on the Society of Thoracic Surgeons (STS) score.

Clinical, electrocardiographic, and echocardiographic data were extracted retrospectively, and every patient had a baseline electrocardiogram (EKG) and echocardiogram done before TAVR. Clinical variables studied included age, gender, body mass index (BMI), STS score, history of hypertension, diabetes, prior myocardial infarction, heart failure with different New York Heart Association functional classes (NYHA Class), atrial fibrillation or flutter, smoking, chronic lung disease, and renal disease requiring dialysis.

Echocardiographic variables included left ventricular internal diameter (LVID) measured at systole (LVIDs) and diastole (LVIDd) and ventricular septal wall thickness.

Outcome comparison

The primary outcome was pacemaker implantation.

Statistical analyses

Patients were divided into four categories based on their BMI: underweight (BMI < 25; 67 patients), normal (BMI: 25~ < 30; 87 patients), overweight (BMI: 30~ < 35; 60 patients), and obese (BMI ≥ 35; 55 patients). Baseline characteristics and clinical data were compared among the groups. Continuous data were represented as mean ± standard deviation (SD) and categorical data as proportions. T-test was used to compare continuous variables; the Chi-square test and adjusted logistic regression were used for categorical variables.

For intensive care unit (ICU) hours and length of stay, a generalized linear model with log link and Poisson distribution were used.

The key covariate was a PPM status variable. Common covariates for adjusted analysis included age, male, smoking status, STS score, BMI, LVIDs, LVIDd, septal wall thickness, valve type, valve size, access type, prior NYHA, chronic lung disease, diabetes, dialysis, prior myocardial infarction (MI), prior two-week heart failure (HF), hypertension, atrial fibrillation/flutter, and conduction defect. When running the logistic analysis of the PPM outcome, the following variables were omitted due to collinearity: valve size, access type, and dialysis.

All calculations were performed using Stata 12 (StataCorp LLC, College Station, Texas, USA) and a p-value of less than 0.05 was considered statistically significant.

## Results

Baseline characteristics

There were 269 patients included in this study (Table 1). Of these patients, 50.6% were males, the average age was 79.5 ± 8.7 years, and the mean STS score was 6.2. Permanent pacemaker implantation after TAVR was seen in 17 (6.3%) of these patients. The average time to pacemaker implantation following TAVR was 1.3 days.

**Table 1:** Demographics and Baseline Characteristics # of sample (proportion% by column), Mean (SD: standard deviation) A fib: atrial fibrillation; Cr: creatinine; Hb: hemoglobin; HF: heart failure; LVIDd: left ventricular internal diameter diastolic; LVIDs: left ventricular internal diameter systolic; MI: myocardial infarction; NYHA: New York Heart Association; O_2_: oxygen; PPM: permanent pacemaker; STS Score: Society of Thoracic Surgeons Score

Variables	All sample (N=269)	PPM (N=17)	No PPM (N=252)	P value*
Age	79.5 (8.7)	79.5 (8.7)	80.6 (8.7)	0.591
Male	136 (50.6%)	11 (64.7%)	125 (49.6%)	0.228
Smoker	13 (4.8%)	2 (11.7%)	11 (4.4%)	0.169
Hypertension	246 (91.4%)	14 (82.3%)	232 (92.1%)	0.166
Diabetes	120 (44.6%)	7 (41.2%)	113 (44.8%)	0.769
Home O_2_	11 (4.1%)	1 (5.9%)	10 (3.9%)	0.700
Immunosuppression	20 (7.4%)	1 (5.9%)	19 (7.5%)	0.801
Prior MI	85 (31.6%)	6 (35.3%)	79 (31.4%)	0.735
Prior HF	39 (14.5%)	3 (17.6%)	36 (14.3%)	0.703
A fib/flutter	99 (36.8%)	8 (47.1%)	91 (36.1%)	0.365
Conduction Defect	130 (48.3%)	15 (88.2%)	115 (45.6%)	0.001
Conscious sedation	178 (66.2%)	11 (64.7%)	167 (66.3%)	0.895
Body Mass Index	30.3 (7.7)	32.9 (6.4)	30.1 (7.7)	0.037
Underweight (< 25)	67 (24.9%)	2 (11.8%)	65 (25.8%)	
Normal (25~ < 30)	87 (32.3%)	2 (11.8%)	85 (33.7%)	
Overweight (30~ < 35)	60 (22.3%)	7 (41.2%)	53 (21.0%)	
Obesity ( ≥ 35)	55 (20.5%)	6 (35.3%)	49 (19.4%)	
Prior NYHA 4 category				0.966
I	2 (0.7%)	0 (0.0%)	2 (0.8%)	
II	28 (10.4%)	2 (11.7%)	26 (10.3%)	
III	121 (44.9%)	7 (41.2%)	114 (45.2%)	
IV	118 (43.8%)	8 (47.1%)	110 (43.6%)	
Prior NYHA 2 category				0.934
I-II	30 (11.1%)	2 (11.8%)	28 (11.1%)	
III-IV	239 (88.8%)	15 (88.2%)	224 (88.9%)	
Chronic lung disease				0.951
None	154 (57.3%)	9 (52.9%)	145 (57.5%)	
Mild	53 (19.7%)	4 (23.5%)	49 (19.4%)	
Moderate	41 (15.2%)	3 (17.6%)	38 (15.1%)	
Severe	21 (7.8%)	1 (5.9%)	20 (7.9%)	
STS score	6.2 (5.9)	6.7 (5.9)	6.2 (4.9)	0.687
Hb pre-procedure	12.1 (1.7)	12.9 (1.9)	12.0 (1.6)	0.021
Cr pre-procedure	1.3 (0.9)	1.2 (0.7)	1.3 (0.9)	0.689
LVIDs	3.2 (0.8)	3.1 (0.8)	3.2 (0.8)	0.669
LVIDd	4.6 (0.7)	4.8 (0.9)	4.6 (0.7)	0.371
Septal wall				0.414
< 1.1	52 (19.3%)	2 (11.8%)	50 (19.8%)	
≥ 1.1	217 (80.7%)	15 (88.2%)	202 (80.2%)	

Comparison of BMI

Patients receiving permanent pacemaker (PPM) implantation showed a statistically higher BMI than patients not receiving PPM (PPM: 32.9 ± 6.4 vs. non-PPM: 30.9 ± 7.7; p = 0.037). Patients were divided into four categories based on their BMI: underweight (BMI: < 25; 67 total patients), normal (BMI: 25 - 30; 87 patients), overweight (BMI: 30 - 35; 60 patients) and obese (BMI: ≥ 35; 55 patients). When comparing PPM implantation between BMI classes, the pacemaker implantation rate was significantly higher in patients with a BMI > 30 (13 vs. 4, p = 0.037).

Other clinical variables

There was a comparatively higher proportion of patients receiving PPM who showed a conduction defect compared to non-PPM patients (88.2% vs. 45.6%; p = 0.001). On average, PPM patients also had higher hemoglobin prior to TAVR (12.9 vs. 12.0; p = 0.021). Other variables were statistically significant.

Regression variables

After logistic linear regression adjustment for other variables (Table 2), the odds of receiving a PPM after TAVR were statistically higher in patients who were overweight (odds ratio (OR): 12.77, p = 0.024; confidence interval (CI): 1.39 - 17.25) and obese (OR: 15.02, p = 0.036, CI: 1.19 - 19.92). The odds of PPM implantation in underweight patients was not statistically significant (p = 0.603).

**Table 2:** Adjusted Logistic Regression Result (Outcome: PPM, n = 255) Valve size of 20 mm (14 observations) omitted due to no observation in the PPM group A fib: atrial fibrillation; BMI: body mass index; Cr: creatinine; Hb: hemoglobin; HF: heart failure; LVIDd: left ventricular internal diameter diastolic; LVIDs: left ventricular internal diameter systolic; MI: myocardial infarction; NYHA: New York Heart Association; O2: oxygen; PPM: permanent pacemaker; STS Score: Society of Thoracic Surgeons Score

Covariates	Odds Ratio	P-value	95% Confidence Interval	
Age	1.03	0.623	0.92	1.15
Male	0.09	0.088	0.01	1.44
Smoker	22.56	0.068	0.80	640.04
STS score	1.08	0.460	0.88	1.33
BMI (Ref: Normal)				
Underweight	2.01	0.603	0.14	27.92
Overweight	12.77	0.024	1.39	17.25
Obese	15.02	0.036	1.19	19.92
Hb pre-procedure	1.73	0.025	1.07	2.79
Cr. pre-procedure	0.68	0.552	0.19	2.40
LVIDs	0.41	0.311	0.07	2.30
LVIDd	3.12	0.246	0.46	21.22
Septal wall ≥ 1.1 (Ref: < 1.1)	0.68	0.728	0.08	5.82
Valve size (Ref: 23 mm)				
26 mm	8.34	0.078	0.79	88.32
29 mm	20.21	0.046	1.06	385.36
Moderate anesthesia	0.64	0.642	0.10	4.26
Prior NYHA III-IV	0.57	0.585	0.07	4.32
Chronic lung disease (Ref: None)				
Mild	1.48	0.675	0.24	9.31
Moderate	1.22	0.853	0.15	10.07
Severe	0.01	0.096	9.3E-05	2.12
Diabetes	0.72	0.665	0.16	3.21
Home O_2_	1.11	0.944	0.06	22.52
Immunosuppression	1.37	0.821	0.09	20.33
Prior MI	1.18	0.836	0.25	5.65
Prior HF	2.33	0.448	0.26	20.75
Hypertension	0.19	0.168	0.02	2.00
A fib/flutter	2.74	0.160	0.67	11.13
Conduction Defect	14.40	0.009	1.97	105.13

Patients with higher hemoglobin prior to TAVR showed slightly greater odds of receiving a permanent pacemaker (OR: 1.73, p = 0.025, CI: 1.07 - 2.79). Patients who received a 29 mm-sized valve during TAVR had significantly greater odds of receiving a PPM after the procedure (OR: 20.21, p = 0.046, CI: 1.06 - 385.36). Patients with a conduction defect showed greater odds of receiving a PPM as well (OR: 14.40, p = 0.009, CI: 1.97 - 105.13). No other variables after regression analysis were statistically significant.

## Discussion

TAVR has proven to be a safe alternative to surgical valve replacement in intermediate to high-risk patients with symptomatic severe aortic stenosis. Numerous publications have outlined risk factors for post-intervention mortality and morbidity, and several reports an “obesity paradox” in which complications decline linearly with increasing body mass index (BMI) [13-14]. One complication of TAVR is conduction abnormalities requiring permanent pacemaker implantation with a previously quoted risk of 5% - 25% [1]. Review of prior literature identified a gap in knowledge regarding specifically to the relationship between BMI and necessity of PPM placement post-TAVR [5].

Our study retrospectively identified 449 patients who underwent TAVR at OSF Saint Francis Medical Center between December 2012 and April 2018. Our facility exclusively utilizes balloon-expandable delivery systems from the Edwards Lifesciences Sapien line of devices in which prior studies have identified the lower risk of post-implant conduction defects around 5% [15] when compared to self-expanding device delivery systems around 12% - 39% [16]. To limit confounding, we excluded any valves other than the ESV3 pacemaker (127 patients), an implantable cardiac defibrillator implant prior to the procedure (37 patients), or aborted procedures (16 patients). After exclusion, our sample size was 269 patients. Our cohort compared well to a prior published study with 17 patients (6.3%) requiring pacemaker implantation [1]. Our population was well-distributed based on the standard BMI categorizations and each study group was comprised of 55 - 87 patients (20.5% - 32.3%). Analysis of baseline cohort characteristics revealed that underlying conduction defects (P = 0.001), pre-procedure hemoglobin (P = 0.021), and body mass index (P = 0.037) were statistically significant based on T-test and Chi-squared testing. Logarithmic regression analysis determined pre-procedural hemoglobin (P = 0.025), underlying conduction defect (P = 0.009), and 29 mm valve (P = 0.046) reached statistical significance. Pre-procedural anemia may indicate greater overall comorbidity.

The primary outcome of our study was statistically significant with increased BMI > 30 being associated with increased PPM implantation need (P = 0.037). The overweight group (BMI: 30 - 34.9) was associated with an odds ratio of 12.77 (P = 0.024, CI 1.39 - 17.25) and obese classification (BMI > 35) had an odds ratio of 15.02 (P = 0.036, CI 1.19 - 19.92). Our study results are contrary to findings in prior studies which have identified underweight classification (BMI < 25) as being associated with overall increased complication rates [10]. We postulate that increasing BMI may have associated independent confounders not demonstrated in our study population, for which it was not powered to do so. This is consistent with the growing body of evidence challenging the “obesity paradox,” although, to date, the study insights have been limited [13]. Data have shown that metaplastic and infiltrative changes involving the sinus node, atrioventricular node, right bundle branch, and myocardium adjacent to the atrioventricular ring may lead to cardiac conduction abnormalities [17-18]; whether these changes predispose TAVR patients to a high-degree atrioventricular block and subsequent pacemaker implantation is not well-established. In this regard, our study brings up an interesting finding for future research.

Limitations

The primary identified limitation in our study is the inadequate powering to identify confounding variables. Other limitations relate to the process of determining the necessity for pacemaker implantation at a single center which can vary based on regional and facility protocols. Lastly, BMI is a rather nonspecific predictor of overall fitness/nutrition with a prior study utilizing body surface area (BSA) or alternative indirect nutritional indicators [14].

## Conclusions

Our study is novel in that it specifically identified increasing BMI as a risk factor for the necessity of post-TAVR PPM placement. Our belief is that associated comorbid conditions may confound this result, although this will require further research. Our study, however, adds to the growing evidence that challenges the previously described “obesity paradox” in post-TAVR patients.
